# A Neglected Topic in Neuroscience: Replicability of fMRI Results With Specific Reference to ANOREXIA NERVOSA

**DOI:** 10.3389/fpsyt.2020.00777

**Published:** 2020-08-05

**Authors:** Isabelle Horster, Kathrin Nickel, Lukas Holovics, Stefan Schmidt, Dominique Endres, Ludger Tebartz van Elst, Almut Zeeck, Simon Maier, Andreas Joos

**Affiliations:** ^1^ Department of Psychosomatic Medicine and Psychotherapy, University Medical Center, University of Freiburg, Freiburg, Germany; ^2^ Department of Psychiatry and Psychotherapy, University Medical Center, University of Freiburg, Freiburg, Germany; ^3^ Department of Psychosomatic Medicine and Psychotherapy, Ortenau Klinikum, Offenburg, Germany

**Keywords:** replicability, anorexia nervosa, food, functional magnetic resonance imaging (fMRI), neurobiology

## Abstract

Functional magnetic resonance imaging (fMRI) studies report impaired functional correlates of cognition and emotion in mental disorders. The validity of preexisting studies needs to be confirmed through replication studies, which there is a lack of. So far, most replication studies have been conducted on non-patients (NP) and primarily investigated cognitive and motor tasks. To fill this gap, we conducted the first fMRI replication study to investigate brain function using disease-related food stimuli in patients with anorexia nervosa (AN). Using fMRI, we investigated 31 AN patients and 27 NP for increased amygdala and reduced midcingulate activation when viewing food and non-food stimuli, as reported by the original study (11AN, 11NP; Joos et al., 2011). Similar to the previous study, we observed in the within group comparisons (food>non-food) a frontoinsular activation for both groups. Although in AN the recorded activation clustered more prominently and extended into the cingulate cortex. In the between-group comparisons, the increased amygdala and reduced midcingulate activation could not be replicated. Instead, AN showed a higher activation of the cingulate cortices, the pre-/postcentral gyrus and the inferior parietal lobe. Unlike in the initial study, no significant differences between NP>AN could be observed. The inconsistency of results and the non-replication of the study could have several reasons, such as high inter-individual variance of functional correlates of emotion processing, as well as intra-individual variances and the smaller group size of the initial study. These results underline the importance of replication for assessing the reliability and validity of results from fMRI research.

## Background

Anorexia nervosa (AN) usually affects young women and shows high persistence rates of around 50% (1). Furthermore, it has the highest mortality of all mental disorders (2). The etiology is largely unknown, although an interplay of genetic and environmental factors is assumed (3). The AN pathophysiology consists largely of reduced weight, fear of weight gain and a distorted body perception, as well as a cognitive preoccupation with body and food related issues. For this reason, functional magnetic resonance imaging (fMRI) studies have focused on paradigms with disease-related food and body stimuli to investigate the neuronal correlation of the disorder.

The first fMRI study in AN with visual food cues (six patients, six non-patients (NP)) described greater activation of anterior cingulate cortices (ACC), left insular, and amygdala-hippocampal regions (4). Fourteen years later, a meta-analysis across nine studies applying food cues, reported increased activation of frontocingular cortices and lower activation of the parietal brain (5). However, the design and the results differed between the included studies. Three further reviews confirmed these inconsistencies (6–8) and therefore conclusions remain questionable. None of the studies were confirmed by replication, so the reported findings should not yet be regarded as established scientific knowledge.

The necessity of replications is not only increasingly recognized in the neurosciences, but in the entire scientific community (9–12). The awareness of a general lack of data replication in science, also referred to as a “reproducibility/replicability crisis” (13–16), has emerged in particular during the last decade (17). Although it is generally recognized that the replication and reproduction of scientific claims is essential in scientific research, the deficit of replications persists (9). Furthermore, there is no general agreement on the definition or directives of replication procedures (9, 16, 18, 19). The *Committee on Reproducibility and Replicability in Science* (9) suggested the following definition: “Reproducibility is obtaining consistent results using the same input data, computational steps, methods, and code, and conditions of analysis. (…) Replicability is obtaining consistent results across studies aimed at answering the same scientific question, each of which has obtained its own data. Two studies may be considered to have replicated if they obtain consistent results given the level of uncertainty inherent in the system under study.” Other studies in the field also refer to this definition (15, 17, 20) and this publication adheres to it, too. In addition to exact definitions, the precise description of study protocols, data, and results is of importance (21). Replication serves the validation of exploratory results and therefore the transition from exploratory data into knowledge, to generate confirmable and generalizable principles (9).

There have been some replication efforts in the field of fMRI, but the studies are largely limited to NP and to motor and cognitive tasks (15, 17, 22, 23). However, Bennett and Miller (24) strongly assume that factors influencing the ability of replication (i.e., variance) are larger in emotional paradigms and in clinical populations, including eating disorders (25). Furthermore, low sample sizes, low power, and low effect-sizes, which reduce replicability, have been generally reported in the field of fMRI research (26–28). If replication attempts failed with sample sizes of 15–30, as a consequence of low power and low effect-sizes, this would have profound influences on planning further studies with respect to number of participants and study set-ups (29).

Against this background, the objective of the present study was to replicate for the first time an fMRI study in AN using visual food and non-food stimuli. Our aim was to replicate the original study (30) with the same research question in a larger but similar sample, using the identical study design and closely following the fMRI and analysis protocol.

In the original study (30), both AN (N=11) and NP (N=11) showed an involvement of frontoinsular and ACC areas when comparing food>non-food pictures (within-group effects) (**Figure 2A**). Comparing the two groups, AN had elevated blood oxygenation level dependent (BOLD) responses of the right amygdala and less activation in midcingulate cortices (MCC).

We assume that (1) there will be different neural correlates of the food-stimuli in AN compared to NP, uncovering disease-related responses, (2) that within-group data of food>non-food pictures will show an involvement of frontoinsular and cingulate cortices, and (3) between-group data will reveal elevated BOLD responses of the right amygdala and decreased activation in midcingulate cortices (MCC) in AN compared to NP similar to our earlier results.

In addition, we assessed emotional reactions to the stimuli by rating the images after scanning.

## Materials and Methods

This study was part of a multimodal MRI study, which assessed structural, metabolic and other functional data [see, e.g., (31–36)]. We replicated the aforementioned food paradigm with 31 AN and 27 NP.

In the following, we first describe Material and Methods of the current study and point towards differences with the earlier study in the second section.

### Current Replication Study

#### Sample and State of Participants

For sample description see **Table 1**. All participants were studied in the second half of the menstrual cycle or the equivalent stage with estrogen and progesterone when taking oral contraception in the current investigation. All participants were offered a standardized breakfast before scanning. Caloric intake was (expectedly) lower in the AN group (**Table 1**). Of the 31 AN, 28 were diagnosed with a restrictive and 3 with a binge-eating/purging subtype.

**Table 1:** Clinical characteristics of anorexia nervosa (AN) and non-patients (NP).

	*Anorexia Nervosa(N=31)*		*Non-Patients(N=27)*		*T-Test*	
					*t-score*	*p-value*
	Mean	SD	Mean	SD		
*Age (years)*	24.0	4.4	23.6	3.0	0.44	0.659
*Duration of illness (years)*	6.6	3.8	-	-		
*Current BMI (kg/m^2^)*	16.2	1.4	22.1	2.2	-11.97	<.001
*Lowest-Lifetime BMI (kg/m^2^)*	14.8	1.5	20.9	1.8	-11.08	<.001
*EDI—total score*	61.8	9.3	44.6	3.1	9.19	<.001
*EDI—drive for thinness (t values)*	83.5	19.6	44.6	6.4	9.85	<.001
*EDI—body dissatisfaction (t values)*	61.7	12.7	46.6	8.0	5.31	<.001
*BDI-II*	21.5	10.5	2.3	2.7	9.2	<.001
*EDE total score*	3.3	1.1	0.4	0.3	13.53	<.001
*MWT-B*	28.4	5.2	28.0	4.3	0.34	0.736
*Caloric intake at breakfast*	142.3	157.5	386.3	85.7	-7.12	<.001
*STAI-state*	38.7	6.6	32.8	4.8	3.83	<.001
*STAI-trait*	45.5	7.7	29.3	6.8	8.39	<.001

BDI-II, Becks Depression-Inventory-2; BMI, Body-Mass-Index; EDE, Eating Disorder Examination Interview; EDI-2, Eating Disorder Inventory-2; kg, kilogram; m2, square meter; MWT-B, Multiple-Choice-Vocabulary-Intelligence Test - German for Mehrfachwahl-Wortschatz-Test-Version (37); SD, standard deviation; STAI, State-Trait Anxiety Inventory.

#### Paradigm Presentation

The same visual food cues as in the previous study were presented in a block design showing 10 consecutive pictures of food followed by 10 consecutive non-food pictures per block – with a duration of 3 s per picture. As mentioned in Joos et al. (30) some of the stimuli have been created by ourselves while others were kindly provided by R. Uher and colleagues (38).

Five blocks of each condition were presented. Examples of the stimuli used can be found in **Supplement 1**.

The instruction was identical to the previous study: participants should watch the pictures attentively (30).

#### MRI Data Acquisition and Preprocessing

A T1-weighted MPRAGE sequence was recorded as an anatomical reference (repetition time (TR): 2300ms, echo time (TE): 2.98ms, flip angle (FA): 90°, field-of-view (FOV): 240*256 mm, 176 slices, voxel size: 1x1x1 mm) using a Siemens 3T PRISMA Magnetom (Erlangen, Germany) equipped with a 20-channel head coil. The T1-weighted sequence was followed by the recording of 159 functional echo-planar T2*-weighted (EPI) images (TR: 2,500 ms, TE: 30 ms, FA: 90°, FOV: 192*192 mm, 38 slices, voxel size: 3x3x3 mm, interleaved). All EPI volumes were automatically rigid-body transformed to correct for head motion and a distortion correction algorithm was applied (39).

The statistical parametric mapping software SPM12 [Welcome Trust Centre of Imaging Neuroscience, London; for details, see (40)] was applied for the preprocessing and statistical analyses of the functional data. The first two volumes of each run were disregarded as so-called dummy scans, an artifact detection algorithm (ArtRepair toolbox, SPM) was applied to detect head motion and spiking artifacts. The realignment to the first volume of the raw functional images that were not motion corrected, was done to generate six head motion parameters (rotation and translation in x, y, z direction). To correct for influences of head motion those parameters were entered in the statistical first-level analysis as regressors of no interest. Using the anatomical MPRAGE image the remaining motion corrected images were spatially normalized with the Montreal National Institute (MNI) reference system followed by the smoothing of the functional images using a three-dimensional isotropic Gaussian kernel (8 mm full width at half maximum) to increase the signal-to-noise ratio and to compensate for inter-individual differences in location of corresponding functional areas. To remove low frequency artifacts across the time-series we applied a high-pass filter (128 s).

#### Statistical Analyses

Psychometric and behavioral data were assessed by two-sample t-test with a level of significance of p<0.05.

For functional data a linear regression model (general linear model [GLM]) with six regressors, modeling the head motion parameters of the realignment procedure, was fitted to the signal time courses of each voxel for each participant. The food and nonfood regressors were fitted with a canonical hemodynamic response function.

##### Whole Brain Second Level Analysis Replicating the Original Study

The resulting beta estimates for the two regressors were fed into a voxel-wise group-level random effects analyses using SPM’s ‘‘full factorial’’ model with the factors condition (food and nonfood) and group (AN, NP) (30). Two different SPM t-contrasts of differential activation towards food versus nonfood condition were calculated for the comparisons AN(Food>non-food) >/< NP(Food>non-food*)*. Bar graphs of activity were generated using the rfx plot as described by Gläscher (41). For the replication of Joos et al. (30) group activation maps (food versus nonfood) we used for the within-group comparisons a cluster-defining threshold of p_uncorr._<0.001 (> 10 voxels) and for the between-group comparison a cluster-defining threshold of p_uncorr._<0.01 (> 0 voxels). Results were considered significant at p<0.05, corrected for multiple comparisons (Family-wise error corrected (FWE)).

##### Region of Interest-Based Second Level Analysis Replicating the Original Study

In addition to the whole brain analysis, a region of interest (ROI) approach was conducted. As performed by Joos et al. (30), the following ROIs according to the Automated Anatomical Labeling Atlas [AAL; (42)] were used: medial and lateral orbitofrontal cortex (OFC), amygdala, ACC, insula and parietal lobe. Again, data were corrected for multiple comparison applying family wise error correction (p<0.05), as a small volume correction (SVC) for all voxels in the corresponding ROI.

##### Whole Brain Second Level Analysis According to Current Recommendations

Within-group food > nonfood differences were calculated using a one-sample t-test for both the AN and NP group. Further, the food > nonfood contrasts of the two groups were compared in a two-sample t-test. For both analyses the cluster-defining thresholding was set to p_uncorr._<0.001, k ≥ 10 (43–46).

##### ROI-Based Second Level Analysis According to Current Recommendations

A SVC was conducted using the ROIs and the t-statistics described above.

### Methodological Differences to the Original Study

#### Sample and State of Participants

**Figure 1:**
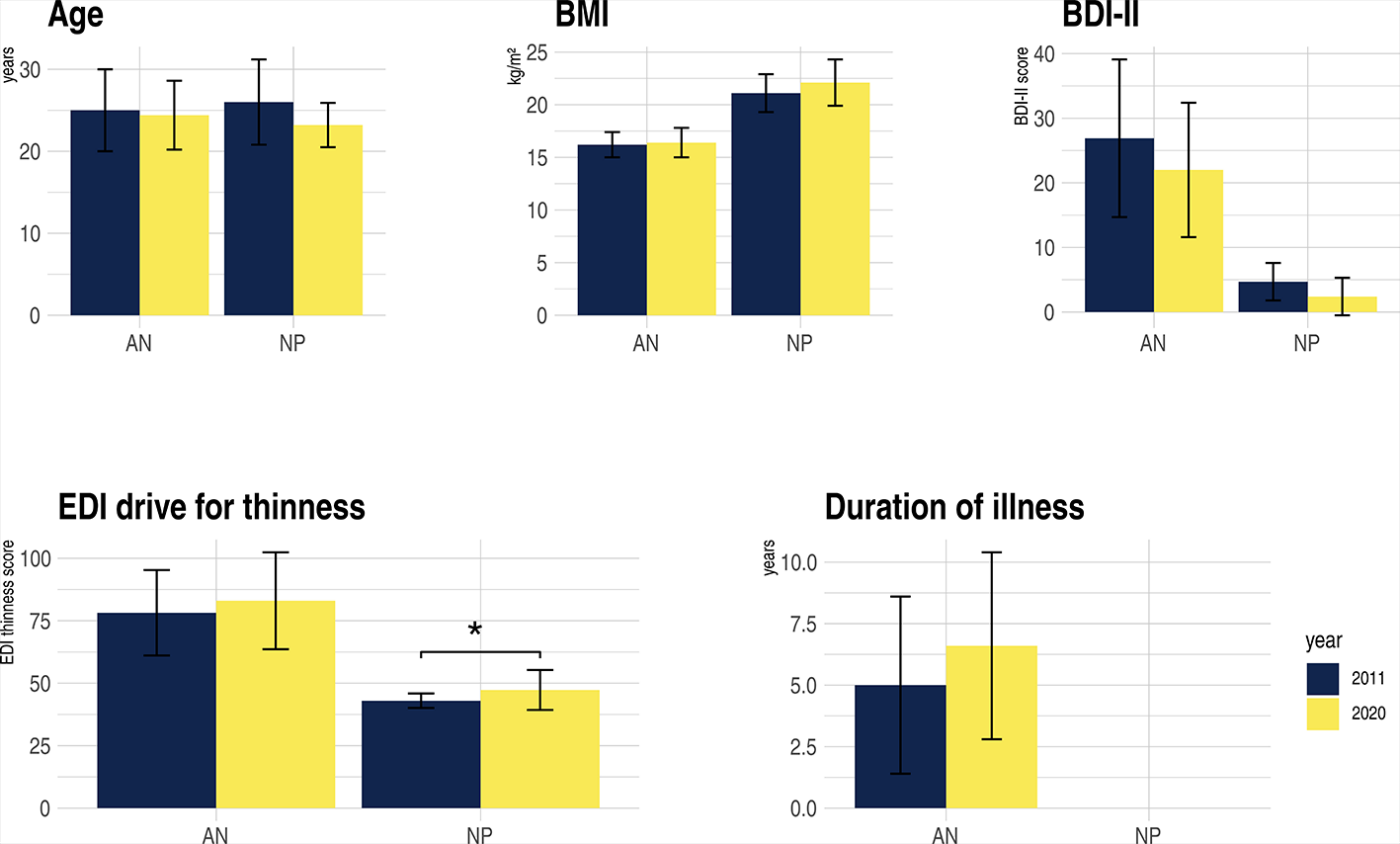
Clinical characteristics of anorexia nervosa (AN) and non-patient (NP), study sample 2011 compared to 2020. BDI-II, Becks Depression-Inventary-2; BMI, Body-Mass-Index; EDI-drive for thinness, Eating Disorder Inventory; kg, kilograms; m^2^, square meter; *p = 0.014. For further clinical characteristics of the replication study see **Table 1**.

The sample size was larger, however clinical characteristics were similar (**Figure 1**). In the earlier study we neither controlled for menstrual cycle nor hormonal contraception, nor was the breakfast standardized (30). Furthermore, the current study was undertaken in the morning, while the former took place in the afternoon hours.

#### Paradigm Presentation

Visual stimuli were now presented with a BOLD Screen system, which has a better contrast and resolution than the rear-projection system used in the Joos et al. (30) study. Additionally, other fMRI data were gathered before the food paradigm, which was not the case in the initial study. In the current study, we used the manikins of the International Affective Picture System (47) assessing the emotional response to the visual stimuli after scanning (outside the scanner) in three dimensions (arousal, valence, dominance), as we used this approach with another paradigm (32) as part of the multimodal study. In the previous study the Likert scale was applied.

#### MRI Data Acquisition and Preprocessing

A comparison of the scanner parameters of the two studies is presented in **Supplement 2**. Due to a scanner upgrade from a Siemens TRIO to a PRISMA system the original MRI parameters could not be adopted. The repetition time (TR) was lowered from 3 to 2.5 s to improve the sampling rate of the BOLD signal. All these changes aimed to increase the signal-to-noise ratio.

Post-processing of the two data sets was always conducted with the SPM standard settings. Yet, there are some differences in the two post-processing pipelines. Joos et al. (30) discarded 10 functional images, while in the current study two dummy scans were discarded in addition to five scans, which were discarded internally by the MR system. In the SPM5 analysis of the initial study the segmentation algorithm for the T1 images differs from the “new segment” procedure used in SPM12, which models the whole head, rather than just the brain. For further details we refer to “SPM: A history” by J. Ashburner (2012, https://doi.org/10.1016/j.neuroimage.2011.10.025).

#### Statistical Analyses

Additionally to the identical second level and ROI analysis replicating Joos et al. (30) a statistical analysis according to current recommendations was conducted (see *Region of Interest-Based Second Level Analysis Replicating Joos et al. (* 30 *)*)

## Results

### Clinical Characteristics

Clinical details are listed in **Table 1**. The AN and NP group of the current study were of the same age and no significant differences were found in the crystalline intelligence test [MWT-B, (30)]. NP had an expectedly higher BMI than AN. Psychopathology showed typically elevated scores of the questionnaires and interviews in AN (**Table 1**). With respect to the standardized breakfast before the measurement, the AN patients consumed fewer calories than the NP. **Figure 1** illustrates the similarities of the clinical characteristics of the original compared to the replication study.

### Subject Rating of Stimuli

Affective ratings of the food stimuli were more aversive for AN (**Supplement 3**). The AN participants evaluated the food pictures more negatively than the NP in terms of valence, but simultaneously triggered a higher arousal in AN.

### Within-Group Activation

**Figure 2:**
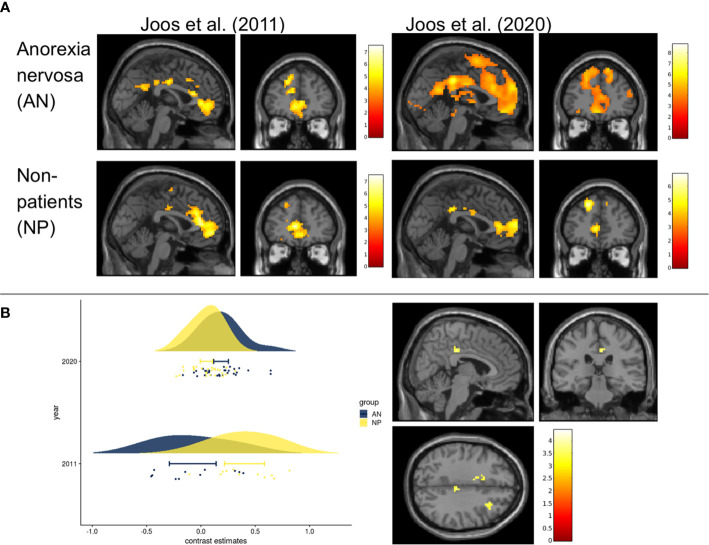
Within group and between group contrasts of the replication study compared to results of Joos et al., 2011 **(A)** Cerebral activation of the within group contrast of anorexia nervosa and non-patients for food>non-food (p _uncorrected <_0.001, k=10, for visualization purposes). Results of Joos et al., 2020 (one-sample t-test) compared to those of Joos et al., 2011 (full-factorial). All slices at MNI coordinates (0, 45, 0) where chosen as in Joos et al., 2011 for a good comparison. Color bars represent the t-scores (white/yellow = high, red = low). Maps from Joos et al., 2011 with kind permission of Elsevier. **(B)** Left Fig. Rain cloud plot of the contrast estimates (food>non-food) in the non-significant midcingulate cortices (MCC) of the replication study (MNI: x=6, y=-28, z=35, with 3mm radius), next to the MCC which derived from the NP>AN contrast reported by Joos et al., 2011 (MNI: x=9, y=-33, z=47). Right Fig: T-maps of the second level analysis (t-test AN(food>non-food) >NP (food>non-food)) according to the current recommendations (cluste-defining threshold p<0.001). Slices where chosen at non-significant peak cluster activity in the right MCC AN>NP (MNI coordinates x=6, y=-28, z=35); p_uncorrected_ <0.001, k>0) Color bars represent the t-scores (white/yellow = high, red = low).

In both groups, increased neuronal activity was found in the frontoinsular region and visual cortex observing the food stimuli compared to the neutral stimuli. In addition, AN showed increased activity of the precuneus, supramarginal, postcentral, and angular gyrus and NP of the superior parietal gyrus (**Figure 2A**, **Supplement 4**).

### Group Comparison

#### Second Level Analysis Replicating the Original Study

Between-group effects yielded higher BOLD signals (AN>NP) in two clusters, one on each hemisphere, including the cingulate cortices, pre-/postcentral gyrus and inferior parietal lobe (IPL) (**Supplement 5**). The contrast NP>AN failed to reveal significant results. In the SVC analyses none of the ROIs showed any group differences.

#### Second Level Analysis According to Current Recommendations

The two-sample t-test with a threshold of p_uncorr._<0.001 did not yield any between-group effects (**Figure 2B**). Also in the SVC analyses no significant group differences emerged in the ROIs.

## Discussion

Our data indicates that within-group effects of food>non-food showed more extensive activation in similar cerebral regions (frontoinsular cortices) in AN and less extensively in NP compared to the previous work (30). Similar patterns of brain activation have been reported in earlier studies that used visual food cues (6). However, when contrasting these activations to NP in the between-group comparison, findings of increased amygdala and decreased MCC activation in AN could not be replicated. In both the current and the previous study (30), as well as in a similar study by Uher et al. (38) AN participants experienced the food stimuli more aversive compared to NP. Therefore, even though the aversive emotions were similar, the neural correlates in the between-group comparison of the studies differed.

The issue of replicability is gaining increased importance in the field of neuroscience, including eating disorders (14, 24, 25). There are several factors that can affect the replicability of results, ranging from the paradigmatic differences to hardware, to intra- and interindividual variances (17). Emotional paradigms seem to be much more critical, particularly in clinical populations (24), which we will discuss in detail below.

In addition to general reasons for poor replicability of studies, such as lack of statistical power, handling of outliers, reporting low p-values or trends (24, 25), and publication biases, the following factors are of particular importance:

1. Compared to within-group statistics, effect-sizes of between-groups in fMRI studies on mental disorders are usually lower (26, 28). From today’s point of view, the original study in particular was conducted with a sample size that was too small, which, considering the relatively small effect sizes resulted in a low power of the study. It is therefore likely that the reported results of the original study were false positive or that at least the effect sizes were overestimated, which increases the likelihood of non-replicability. Since the replication study also failed to detect any group differences when applying conservative thresholds, only studies with a large sample size will have enough power to detect the probably rather weak effects. The only way to deal with relatively small effect-sizes is to increase sample size, and efforts such as those of the ENIGMA (Enhancing Neuro Imaging Genetics through Meta-Analysis) consortium pooling data from many sites (17, 25). Furthermore, larger sample sizes lead to an increase in power (17, 23, 48). As pointed out in several recent papers (43–45, 49), cluster-defining thresholds were often set too low, e.g., p_uncorr_. < 0.01, which increases the risk of false-positive results. However, this procedure was common at the time of planning the initial study (Woo et al. (44) call it “endemic”). No significant group differences emerged when applying the currently recommended strict thresholds (for further details see, e.g., 42, 43, 44).
2. Heterogeneity across participants is an important confounder, not only in patients but also NP. In our two studies many factors are comparable (age, BMI, duration of disorder, psychopathology, in particular drive for thinness, and most being of the restrictive subtype, depression scores and perception of food pictures are more aversive in AN compared to NP – **Figure 1**), while other confounding genetic, environmental and stochastic factors are difficult or even impossible to account for. Some of these factors likely have larger effect-size than the investigated condition itself (50). Studies with small sample sizes might report results that are based on the effect of uncontrolled variables towards the dependent one (48). This also carries the risk of false-positive results due to sampling error. False-positive results may thus lead into a wrong direction, or even worse, may hinder detecting the real pathophysiological mechanisms (51).
3. Similarly, heterogeneity within participants can impact replicability. Depending on the paradigm, different intrinsic factors can influence the BOLD signal. The current study was controlled for effects of daytime (morning) and state of hunger (standardized meal beforehand), which was not the case in the original study. In the morning, hormonal levels like cortisol are higher; similarly, sex hormones exert cerebral effects (25), which was controlled for in the latter but not in the former study. This also increases the probability of false-positive results of the original study.
4. Heterogeneity across study sites arise from different sources. In addition to different fMRI protocols, scanner hardware and image post-processing pipelines, differences in experimental setup (instructions, interaction with the experimenter, order of tests) have an impact (25). In the current study, participants were subjected to other MRI paradigms before the food paradigm was assessed. In the former study participants started with the food paradigm. While an identical post-processing pipeline was used, fMRI protocols and the scanner hardware differed (see material and methods 2.2., **Supplement 2**). Still, person-related variance seems to be clearly greater than site-related variance (24, 25, 50).

### Limitation

The cluster-defining threshold of p<0.01 and the full-factorial model in the between group comparisons are a limitation of the former study. This approach is not in line with the current recommendations. In order to ensure the replication of the former study, we applied a methodology as similar as possible, starting with the same statistical between-group analysis and followed by a statistical analysis according to the current recommendations. Despite being considerably larger than in the previous study, the sample size was still too small. As recent studies point out, due to low effect sizes in the field of fMRI research sample sizes of 100 (52) or even more participants would be necessary (29) to achieve a sufficient power for many effects. Considering these issues, it will be difficult to recruit enough participants in diseases with low prevalence and often low motivation like AN within single center trials; also, costs and efforts will be very high.

Modern scanner hardware seem to influence variability only modestly (24, 25). Differences between SPM5 and SPM12 are mainly in the improved segmentation process and should explain only a minor part of the variance (53).

Another issue discussed in the literature is temporal and spatial stability of fMRI which is influenced by the sensitivity of detecting short-term metabolic changes and neuromodulatory effects (54). Therefore, Logothesis (54) points towards the fact that the fMRI signal of neuromodulatory effects may exceed the signals of purely task-related neuronal activity. This influences not only temporal but also spatial stability. Furthermore, temporal differences in attention, motivation, and excitement, as well as different cognitive strategies for task accomplishment, or changes in cognitive strategy when working on a task, can significantly influence neural activity in response (24). In the original as well as in the replication study, we performed a cross-sectional analysis with a onetime measurement of the participants. Therefore, we cannot assess the influences of short-term metabolic changes and neuromodulatory effects on the BOLD-signals measured. Especially task fMRI studies and within those particularly clinical populations with emotional paradigms seem to be influenced by temporal and spatial instability (24, 29).

## Conclusion

In the replication study, we were not able to identify elevated BOLD responses of the right amygdala and decreased activation in midcingulate cortices (MCC) in AN compared to NP in the between-group analysis and therefore could not replicate the original study (30). As expected, we and other authors (24, 25) assume that human influences (inter- and intra-individual variances) are greater than most other factors and more difficult to control, especially in emotional tasks and in clinical populations.

Nevertheless, like most other fMRI studies that examine neural correlation of food compared to non-food stimuli (5–8), we found differences between AN and NP while processing food versus non-food stimuli applying the second level analysis replicating Joos et al. (30). The increased activation in AN>NP in the MCC together with the pre-/postcentral gyrus has also been reported by others: an increased cingulate activation was described by Ellison et al. (4) and Gizewski et al. (55), an pre-/postcentral gyrus activation by Boehm et al. (56). No increased IPL activation has been mentioned in AN, while a decreased IPL activation could be observed in three studies (38, 57, 58). Of those studies included in the meta-analysis and reviews only Kerr et al. (59) reported no differences between AN and NP for food versus non-food. Due to the heterogeneity of the previous results, no definitive conclusions can yet be drawn from these studies. Further, second level analysis according to current recommendations with a threshold of p_uncorr._<0.001 revealed neither between-group effects in the whole brain nor in the ROI analysis.

We aim to understand the cerebral pathophysiology of AN including the pathological eating behavior and maladaptive eating behavior. For valid and reliable conclusions of functionally altered brain regions, replications of fMRI studies examining neural processing of disease-specific food stimuli are paramount. As noted by others, study protocols as well as samples should be precisely described in order to be able to replicate and disentangle possible influences (17, 21, 24, 25). Likely, replication studies should be performed with larger sample sizes to increase the statistical power (26–28). Additionally, longitudinal studies or studies with repeated sessions of the same participants can be used to create replicability maps (17), which can improve the temporal and spatial stability. Besides the lack of replications, reproductions are necessary as well. Reproduction, i.e., the exact re-analysis of the same data (see *Background*), is a necessary step to establish stable data analysis pipelines and therefore also an important prerequisite for replication studies (60).

The issue of replication has been largely neglected in the past and is now increasingly coming into focus. It is of great importance to carefully control and/or describe modifying factors such as hardware, processing pipelines, statistics, experimental setups and clinical descriptions. Since almost all fMRI studies so far have not undergone replication, the validity of most findings in this field can be challenged.

## Data Availability Statement

The raw data supporting the conclusions of this article will be made available by the authors, without undue reservation. T-maps of the within and between group comparisons are available at: https://identifiers.org/neurovault.image:395600.

## Ethics Statement

The studies involving human participants were reviewed and approved by Ethics commission of the Albert-Ludwig-University Freiburg (Nr. EK-Freiburg 520/13). The patients/participants provided their written informed consent to participate in this study.

## Author Contributions

Planning of the study: AJ, LT, and AZ. AJ is principal investigator of the DFG project JO 744-2/1. Recruitment and psychosomatic assessment: AJ, SM, LH, and AZ. Measurement and data analysis: IH, AJ, SM, LH, KN. Writing: IH, AJ, SM, SS, KN, and DE. Proof reading: AJ, SM, IH, SS, LH, KN, DE, LT, and AZ. All authors contributed to the article and approved the submitted version. They agreed to be accountable for all aspects of the work.

## Funding

The project was funded by the German Research Foundation (DFG Ref: JO 744-2/1). The article processing charge was funded by the University of Freiburg in the funding program Open Access Publishing.

## Conflict of Interest

The authors declare that the research was conducted in the absence of any commercial or financial relationships that could be construed as a potential conflict of interest.
