# Erratum: A Neglected Topic in Neuroscience: Replicability of fMRI Results With Specific Reference to ANOREXIA NERVOSA

**DOI:** 10.3389/fpsyt.2020.599179

**Published:** 2020-09-04

**Authors:** Frontiers Production Office

**Affiliations:** Frontiers Media SA, Lausanne, Switzerland

**Keywords:** replicability, anorexia nervosa, food, functional magnetic resonance imaging (fMRI), neurobiology

Due to a production error, there was a mistake in **Table 1** as published. The footnote text was incorrectly placed and there was an error in the column names. The corrected **Table 1** appears below. The publisher apologizes for this mistake.

The original version of this article has been updated.

**Table 1:** Clinical characteristics of anorexia nervosa (AN) and non-patients (NP).

	*Anorexia Nervosa(N=31)*		*Non-Patients(N=27)*		*T-Test*	
					*t-score*	*p-value*
	Mean	SD	Mean	SD		
*Age (years)*	24.0	4.4	23.6	3.0	0.44	0.659
*Duration of illness (years)*	6.6	3.8	-	-		
*Current BMI (kg/m^2^)*	16.2	1.4	22.1	2.2	-11.97	<.001
*Lowest-Lifetime BMI (kg/m^2^)*	14.8	1.5	20.9	1.8	-11.08	<.001
*EDI—total score*	61.8	9.3	44.6	3.1	9.19	<.001
*EDI—drive for thinness (t values)*	83.5	19.6	44.6	6.4	9.85	<.001
*EDI—body dissatisfaction (t values)*	61.7	12.7	46.6	8.0	5.31	<.001
*BDI-II*	21.5	10.5	2.3	2.7	9.2	<.001
*EDE total score*	3.3	1.1	0.4	0.3	13.53	<.001
*MWT-B*	28.4	5.2	28.0	4.3	0.34	0.736
*Caloric intake at breakfast*	142.3	157.5	386.3	85.7	-7.12	<.001
*STAI-state*	38.7	6.6	32.8	4.8	3.83	<.001
*STAI-trait*	45.5	7.7	29.3	6.8	8.39	<.001

BDI-II, Becks Depression-Inventory-2; BMI, Body-Mass-Index; EDE, Eating Disorder Examination Interview; EDI-2, Eating Disorder Inventory-2; kg, kilogram; m2, square meter; MWT-B, Multiple-Choice-Vocabulary-Intelligence Test - German for Mehrfachwahl-Wortschatz-Test-Version (37); SD, standard deviation; STAI, State-Trait Anxiety Inventory.

