# Supplementary material for: A Neglected Topic in Neuroscience: Replicability of fMRI Results With Specific Reference to ANOREXIA NERVOSA
Source: Front Psychiatry. 2020 Aug 5;11:777. doi: 10.3389/fpsyt.2020.00777 (PMC7419696; doi:10.3389/fpsyt.2020.00777)
Supplement: Supplementary file 1 [file DataSheet_1.pdf]

Supplement 1

Examples of food and non-food Stimuli

Figure 1

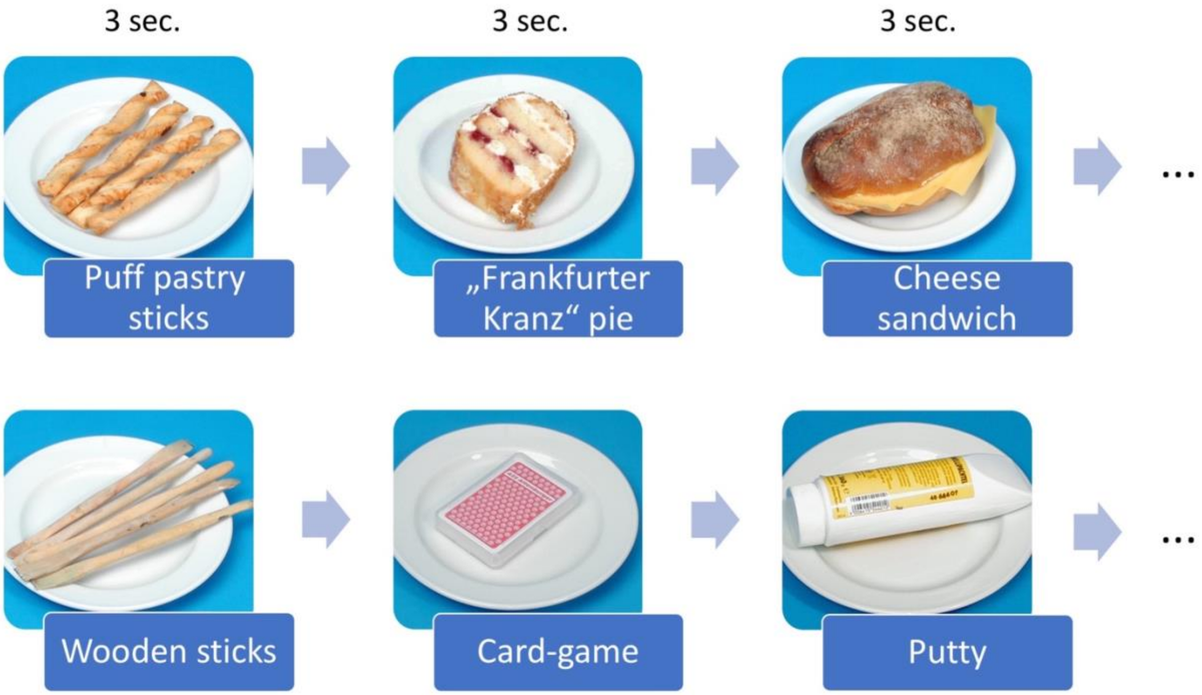

sec. = seconds; first row food stimuli, second row non-food stimuli which were carefully and individually matched for visual complexity based on the ratings by five members of staff in the original study (Joos et al., 2011)

Joos, A.A.B., Saum, B., van Elst, L.T., Perlov, E., Glauche, V., Hartmann, A., Freyer, T., Tüscher, O., Zeeck, A., 2011. Amygdala hyperreactivity in restrictive anorexia nervosa. Psychiatry Res. Neuroimaging 191, 189–195. https://doi.org/10.1016/j.pscychresns.2010.11.008
