# Supplementary material for: A Neglected Topic in Neuroscience: Replicability of fMRI Results With Specific Reference to ANOREXIA NERVOSA
Source: Front Psychiatry. 2020 Aug 5;11:777. doi: 10.3389/fpsyt.2020.00777 (PMC7419696; doi:10.3389/fpsyt.2020.00777)
Supplement: Supplementary file 2 [file DataSheet_2.docx]

Supplement 2

***Differences in the fMRI protocols and the scanner hardware between the current study and Joos et al. (2011)***

Table 1

| **Parameter** | **Current study** | **Joos et al. (2011)** |
| --- | --- | --- |
| **Software** | SPM12 | SPM5 |
| **MR System** | Siemens PRISMA Magnetom | Siemens TRIO Magnetom |
| *T1- weighted* |  |  |
| **TR** | 2300ms | 2200ms |
| **TE** | 2,98ms | 4,91ms |
| **Flip angel** | 9° | 12° |
| **Voxel** | 1x1x1 | 1x1x1 |
| **FOV** | 240*256 mm^2^ | 256*256mm^2^ |
| *T2*- weighted* |  |  |
| **Head coil** | 20 channel | 12 channel |
| **TR** | 2500ms | 3000ms |
| **TE** | 30ms | 30ms |
| **Flip angel** | 90° | 70° |
| **Voxel** | 3x3x3 | 3x3x3 |
| **FOV** | 192mm^2^ | 192mm^2^ |

FOV = field of view, TE = echo time, TR = relaxation time

Joos, A.A.B., Saum, B., van Elst, L.T., Perlov, E., Glauche, V., Hartmann, A., Freyer, T., Tüscher, O., Zeeck, A., 2011. Amygdala hyperreactivity in restrictive anorexia nervosa. Psychiatry Res. Neuroimaging 191, 189–195. https://doi.org/10.1016/j.pscychresns.2010.11.008
