# Supplementary material for: A Neglected Topic in Neuroscience: Replicability of fMRI Results With Specific Reference to ANOREXIA NERVOSA
Source: Front Psychiatry. 2020 Aug 5;11:777. doi: 10.3389/fpsyt.2020.00777 (PMC7419696; doi:10.3389/fpsyt.2020.00777)
Supplement: Supplementary file 3 [file DataSheet_3.pdf]

Supplement 3

Affective ratings of food and non-food stimuli in AN and NP

Experiences were assessed by emotion dimensions according to Lang et al. (2008). [This procedure was adopted due to another emotional paradigm (intimacy: Maier et al. (2019)) of the multimodal study (DFG JO 744/2-1).]

Significance according to t-tests, two-sided.

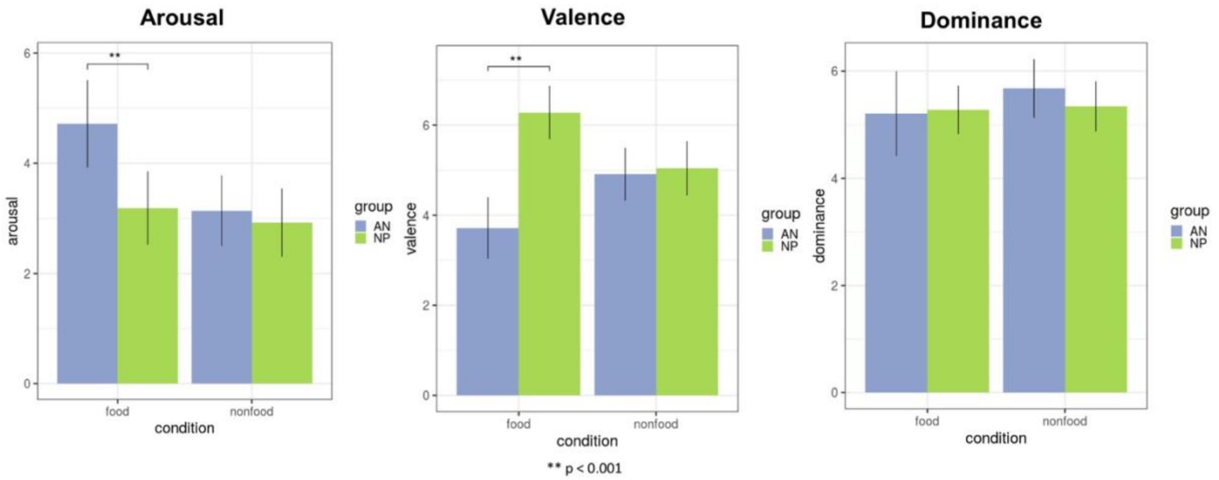

The group of anorexia nervosa (AN) patients experienced increased arousal ($p < 0.001$) during food presentation compared to nonpatients (NP) and decreased valence ($p < 0.001$).

Lang, P.J., Bradley, M.M., Cuthbert, B.N., 2008. International Affective Picture System (IAPS): Affective Ratings of Pictures and Instruction Manual. University of Florida, Gainesville, FL.

Maier, S., Spiegelberg, J., Zutphen, L. van, Zeeck, A., Elst, L.T. van, Hartmann, A., Holovics, L., Reinert, E., Sandholz, A., Lahmann, C., Domschke, K., Glauche, V., Tüscher, O., Joos, A.A.B., 2019. Neurobiological signature of intimacy in anorexia nervosa. Eur. Eat. Disord. Rev. 27, 315–322. https://doi.org/10.1002/erv.2663
