# Supplementary material for: A Neglected Topic in Neuroscience: Replicability of fMRI Results With Specific Reference to ANOREXIA NERVOSA
Source: Front Psychiatry. 2020 Aug 5;11:777. doi: 10.3389/fpsyt.2020.00777 (PMC7419696; doi:10.3389/fpsyt.2020.00777)
Supplement: Supplementary file 4 [file DataSheet_4.docx]

Supplement 4

Brain activations when contrasting food versus non-food stimuli of AN and NP

Within-group food > nonfood differences were calculated using a one-sample t-test for both the AN and NP group. The cluster-defining thresholding was set to p_uncorr._<0.001, k $\boldsymbol{\geq}$10.

| **­** | **Hemi-sphere** | **Cluster** | **p_corr. cluster_^a^** | **p_corr. peak_^b^** | **Voxels** | **MNI** | | | **T-score ^c^** | **Effect size** |
| --- | --- | --- | --- | --- | --- | --- | --- | --- | --- | --- |
|  |  |  |  |  |  | **x=** | **y=** | **z=** |  |  |
| **Anorexia Nervosa** | | | | | | | | | |  |
| Middle occipital gyrus | L | 1 | 0.000 | 0.000 | 1104 | -18 | -100 | -1 | 8.79 | 1.58 |
| Calcarine fissure | R |  |  | 0.001 |  | 15 | -100 | 2 | 7.35 | 1.35 |
| Lingual gyrus | R |  |  | 0.011 |  | 15 | -88 | -4 | 6.05 | 1.08 |
| Fusiform gyrus | R |  |  | 0.013 |  | 30 | -76 | -7 | 6.01 | 1.08 |
| Fusiform gyrus | L |  |  | 0.04 |  | -30 | -76 | -10 | 5.5 | 0.99 |
| Midcingulate | L | 2 | 0.000 | 0.003 | 5534 | 0 | -31 | 35 | 6.68 | 1.19 |
| Supplementary motor area | L |  |  | 0.019 |  | -12 | 23 | 65 | 5.83 | 1.05 |
| Superior frontal gyrus, medial | L |  |  | 0.025 |  | 0 | 59 | 23 | 5.71 | 1.04 |
| Superior frontal gyrus | R |  |  | 0.026 |  | 15 | 38 | 50 | 5.7 | 1.04 |
| Anterior cingulate gyrus | L |  |  | 0.03 |  | -9 | 14 | 26 | 5.63 | 1.01 |
| Middle frontal gyrus, orbital part | L |  |  | 0.03 |  | -6 | 56 | -7 | 5.63 | 1.01 |
| Precuneus | L |  |  | 0.033 |  | -6 | -52 | 20 | 5.58 | 1.00 |
| Superior frontal gyrus | L |  |  | 0.046 |  | -15 | 59 | 26 | 5.44 | 0.98 |
| Insula | R |  |  | 0.047 |  | 39 | -1 | 2 | 5.43 | 0.98 |
| Midcingulate | R |  |  | 0.047 |  | -3 | -10 | 29 | 5.43 | 0.98 |
| Supramarginal gyrus | R | 3 | 0.000 | 0.016 | 323 | 60 | -16 | 29 | 5.91 | 1.07 |
| Insula | L | 4 | 0.000 | 0.016 | 405 | -33 | -1 | 14 | 5.91 | 1.07 |
| Postcentral gyrus | L | 5 | 0.097 | 0.021 | 80 | -60 | -16 | 26 | 5.79 | 1.03 |
| Angular gyrus | R | 6 | 0.011 | 0.084 | 164 | 48 | -61 | 38 | 5.17 | 0.93 |
| **Non-Patients** | | | | | | | | | |  |
| Calcarine fissure | R | 1 | 0.015 | 0.004 | 118 | 18 | -94 | 5 | 6.91 | 1.32 |
| Middle occipital gyrus | R |  |  | 0.417 |  | 33 | -82 | 8 | 4.57 | 0.88 |
| Superior frontal gyrus, medial | L | 2 | 0.000 | 0.004 | 689 | -9 | 50 | 41 | 6.87 | 1.31 |
| Superior occipital gyrus | L | 3 | 0.000 | 0.007 | 249 | -18 | -91 | 2 | 6.63 | 1.28 |
| Insula | L | 4 | 0.003 | 0.015 | 173 | -36 | -7 | 11 | 6.3 | 1.21 |
| Superior parietal gyrus | L | 5 | 0.002 | 0.025 | 189 | -18 | -67 | 59 | 6.03 | 1.16 |

^a^ Cluster level, corrected for multiple comparisons across the whole brain with Family wise error rate correction ^b^ Peak/ voxel level, corrected for multiple comparisons with Family wise error rate correction (FWER) ^c^ Voxel with peak T-score of the cluster.

Effect size: T-statistics were converted to Cohen’s d coefficients using $Cohens \left( d \right)=\frac{t}{\sqrt{N}}$, *t* is the *t*-test statistic, and *N* is the sample size. For further details see eqn. 2.5.9 (Cohen, 2013, p. 72).

Cohen, J., 2013. Statistical Power Analysis for the Behavioral Sciences. Academic Press.
