# Supplementary material for: A Neglected Topic in Neuroscience: Replicability of fMRI Results With Specific Reference to ANOREXIA NERVOSA
Source: Front Psychiatry. 2020 Aug 5;11:777. doi: 10.3389/fpsyt.2020.00777 (PMC7419696; doi:10.3389/fpsyt.2020.00777)
Supplement: Supplementary file 5 [file DataSheet_5.docx]

Supplement 5

***Second level analysis replicating the study Joos et al., 2011***

For the replication of Joos et al. (2011) we used for the between-group comparison (food versus nonfood) AN versus NP a cluster-defining threshold of p_uncorr._<0.01 (> 0 voxels). Results were considered significant at p<0.05, corrected for multiple comparisons (Family-wise error corrected (FWE)).

Between-group effects yielded higher BOLD signals (AN>NP) in two clusters, one on each hemisphere, including the cingulate cortices, pre-/postcentral gyrus and inferior parietal lobe (IPL)

| **Brain region** | **Hemi-**  **sphere** | **p_corr. cluster_^a^** | **p_corr. peak_^b^** | **Voxels** | **MNI** |  |  | **T-score ^c^** | **Effect size** |
| --- | --- | --- | --- | --- | --- | --- | --- | --- | --- |
|  |  |  |  |  | x= | y= | z= |  |  |
| **AN>NP** |  |  |  |  |  |  |  |  |  |
| Cluster 1 |  |  |  | 780 |  |  |  |  |  |
| MCC | L | 0.001 | 0.384 |  | -12 | 11 | 29 | 3.69 | 0,48 |
| Precentral gyrus | L | 0.001 | 0.836 |  | -33 | -7 | 38 | 3.49 | 0.46 |
| Postcentral gyrus | L | 0.001 | 0.996 |  | -39 | -22 | 41 | 3.02 | 0.4 |
| Middle frontal cortex | L | 0.001 | 0.999 |  | -30 | 47 | 32 | 2.90 | 0.38 |
| Cluster 2 |  |  |  | 521 |  |  |  |  |  |
| IPL | R | 0.01 | 0.320 |  | 36 | -31 | 29 | 3.98 | 0.52 |
| Angular gyrus | R | 0.01 | 0.522 |  | 39 | -52 | 29 | 3.82 | 0.50 |
| Precuneus | R | 0.01 | 0.865 |  | 21 | -49 | 29 | 3.45 | 0.45 |
| Posterior cingulate gyrus | R | 0.01 | 0.961 |  | 12 | -40 | 14 | 3.25 | 0.43 |
| **NP>AN** | no significant results. | | | | | | |  |  |

^a^ Cluster level, corrected for multiple comparisons across the whole brain with Family wise error rate correction ^b^ Peak/ voxel level, corrected for multiple comparisons with Family wise error rate correction ^c^ Voxel with peak T-score of the cluster. Effect size: T-statistics were converted to Cohen’s d coefficients using $Cohens \left( d \right)=\frac{t}{\sqrt{N}}$, *t* is the *t*-test statistic, and *N* is the sample size. For further details see eqn. 2.5.9 (Cohen, 2013, p. 72).

Cohen, J., 2013. Statistical Power Analysis for the Behavioral Sciences. Academic Press.
